# Retraction: Decreased miR-204 in H. pylori-Associated Gastric Cancer Promotes Cancer Cell Proliferation and Invasion by Targeting SOX4

**DOI:** 10.1371/journal.pone.0241451

**Published:** 2020-10-22

**Authors:** The PLOS ONE Editors

Following publication of this article [1] and its subsequent correction [2], concerns have been raised about similarities between images in different figures within the article and also between images in [1] and images in another article by some of the same authors [3], reporting different experiments.

Specifically,

- The miR-204 inhibitor panel of Fig 2B in [1] is the same image as the iNC panel of Fig 2B in [3].
- The miR-204 mimic panel of Fig 2B in [1] is the same image as the mimics panel of Fig 2B in [3].
- The iNC and inhibitor panels of Fig 3D in [1] are the same images as the 7901 LV-miR-203 and LV-control panels Fig 2E in [3].
- In S4C Fig [1] the pLV-NC 24h panel appears similar to the iNC 24h panel in Fig 2C
- In S4C Fig [1] the pLV-SOX4 24h panel appears similar to the miR-204 inhibitor 24h panel in Fig 2C
- In S4C Fig [1] the mimics 24h panel appears similar to the miR-204 mimic 24h panel in Fig 2C
- In S4C Fig [1] the mimics 0h panel appears similar to the NC 0h panel in Fig 2C
- In S4C Fig [1]the pLV-SOX4 0h panel appears similar to the miR-204 inhibitor 0h panel in Fig 2C
- In S4C Fig [1] the pLV-NC 0h panel appears similar to the iNC 0h panel in Fig 2C
- In S4C Fig [1] the mimics +pLV-SOX4 24h panel appears similar to the pLV-NC 24h panel in Fig 6B
- In Fig 2 of [1] the legends for panels B and C are switched.

The authors clarified that the duplicate images in Fig 2B of [1] were included in error, and that the iNC and inhibitor panels were correctly reported in Fig 3D of [1] but incorrectly included in Fig 2E of [3]. The underlying raw image files for this article are not available. Partial underlying chart data was provided by the authors, but the underlying data for charts shown in Figs 2B, 3, 4E, 6C, S2, S3B, S3D and S4 are not available.

In the course of editorial follow up on this article, inconsistencies in ethics oversight documentation were identified, and it has come to light that the collection and use of patient tissue samples in this study did not have specific approval from the Ethical Committee of the First Affiliated Hospital of Nanjing Medical University, contrary to the statement included in the Materials and Methods section of the article.

In light of the above issues, the *PLOS ONE* Editors retract this article [1] due to the lack of ethical oversight for the work and concerns about the reliability of the results reported.

GZ disagreed with the retraction. XY, LL, JS either could not be reached or did not reply directly. GZ stands by the article's findings and apologizes for the issues with the published article.
